# Development of Wall-Coated Open Tubular Columns and Their Application to Nano Liquid Chromatography Coupled to Tandem Mass Spectrometry

**DOI:** 10.3390/molecules28135103

**Published:** 2023-06-29

**Authors:** Natalia G. P. Santos, Deyber A. V. Medina, Fernando M. Lanças

**Affiliations:** São Carlos Institute of Chemistry, University of São Paulo, São Carlos 13566-590, Brazil

**Keywords:** column development, open tubular columns, nano liquid chromatography, mass spectrometry, wall coated open tubular column (WCOT)

## Abstract

This work presents a study on the application of wall open tubular column (WCOT) in liquid chromatography coupled with tandem mass spectrometry. Each process step reports the column preparation method in detail, subdivided into column pretreatment, silanization, stationary phase coating, and immobilization. Then, an evaluation of the parameters that can affect the efficiency of these columns was made. Atrazine, clomazone, and metolachlor were used as probes during this step. Factors such as stationary phase composition, length, internal diameter, stationary phase mass employed, and injection volume were investigated. In addition, with the help of Knox and Poppe graphs, the columns’ performance was evaluated to determine the optimal flow rate and the speed-efficiency relationship, respectively. Based on the results, the best configurations for the WCOT column application to the LC system were defined: length—8 m; inner diameter—25 μm; mass of OV-210—2.5% *m*/*v*; and, injection volume—100 nL. Finally, the optimized WCOT column developed in this work was coupled with a commercially-packed trapping column in the nano liquid chromatography system (nanoLC). In this configuration, more significant results were obtained regarding separation resolution, with Rs = 5.9 achieved for the most retained pair of analytes (clomazone and metolachlor).

## 1. Introduction

Modern liquid chromatography is increasingly moving towards the miniaturization of the technique. This procedure involves investments in resizing the analytical instrumentation to work at micro-LC, capillary-LC, and nano-LC scales. Furthermore, analytical columns have received significant attention aiming at reducing the particle’s size and internal diameter [1]. Therefore, emphasis on the miniaturization of the LC technique started and is directly linked to conventional columns packed with particles by just decreasing the column’s internal diameter. However, other columns, such as monolithic and open tubular (OT), received much less attention despite also being helpful in producing high-quality miniaturized LC columns.

Nota introduced open tubular columns in liquid chromatography in 1970 [2]. Such columns contain a thin layer of stationary phase that covers the inner wall of the capillary tube, leaving its interior empty or “open”. This layer can be of porous or non-porous composition, therefore called a porous layer open tubular column (PLOT) and wall-coated open tubular column (WCOT), respectively [3]. Golay applied these columns in gas chromatography in 1957 [4], which quickly replaced the packed columns, as they presented better separation efficiency under pressure and similar analysis time. Therefore, OT columns in gas chromatography are already well established; however, the scenario for OT-LC is not the same. Unfortunately, studies of these columns applied to liquid chromatography were mainly restricted to the period between the ‘1970s’ and ‘1990s’ [5,6,7,8,9,10], in which there was no suitable instrumentation and miniaturized detectors adequate for OT-LC progress. Consequently, without adequate instrumentation, OT-LC columns lost scientific investments, and packed columns began to gain attention until they reached their current efficiency.

During the early OT-LC period, the design, properties, and performance of these columns were compared to packed columns. According to Hibi [11], the application of OT columns with an internal diameter commonly used in gas chromatography (0.25–0.50 mm i.d.) would cause a drastic reduction in the number of theoretical plates (N) since the diffusion coefficients of the analytes in liquid phases is up to 100 times smaller than in gaseous phases [12]. Consequently, for OT-LC to have an efficiency similar to OT-GC, the internal diameter of these columns must be much smaller. According to studies by Knox and Gilbert, the i.d. in LC should be less than 10 μm [13,14], ideally around 2 μm [15]. This restriction makes producing capillary columns with optimal performance extremely difficult. Recently, Liu’s group [16] reported the development of an OT column with 2.7 cm of effective length and 2 μm i.d., coated with an octadecylsilane film for separating six amino acids by LC-UV-Vis. However, the chromatographic separation obtained still presents a noisy baseline, demonstrating that we still need improvement to obtain high-quality robust OT-LC columns with 2 μm i.d.

The main general advantage of OT columns is excluding the A term from the Van Deemter equation, which accounts for almost half of the band broadening in packed columns. They also have high permeability, leading to lower system pressure and giving the possibility of increasing column length. The main limitation related to these columns is their low sample capacity, considering that the thin stationary phase layer has a small surface area to interact with the analytes. For that reason, limited sample concentrations must be introduced into the column in order to avoid clogging and other undesirable effects [1], requiring a drastic reduction in the dimensions of the sample introduction system (“injector”).

Although OT-LC columns of the PLOT kind have been consistently more explored in the last two decades, particularly in the proteomic analysis, WCOT columns have not received the same attention. Further studies in OT-WCOT-LC are still required to establish a column preparation methodology capable of producing robust columns with smaller internal diameters. Besides, limitations such as load capacity, column clogging, and/or the chromatographic system still need to be overcome. Therefore, this work aims to contribute to a systematic study of OT-LC columns. Here, we developed WCOT-type columns for application in nano-LC coupled to tandem mass spectrometry fitted with electrospray ionization. Columns were coated with liquid stationary phases based on polysiloxane phases commonly applied only in open tubular gas chromatography. Afterward, a systematic study of parameters that can affect the efficiency of this type of column was carried out, including the stationary phase composition, column length, internal diameter, mass of stationary phase, and injection volume. Furthermore, a study coupling WCOT columns with packed trapping columns was also evaluated, aiming to understand several aspects that may contribute to the better performance of these columns in liquid chromatography.

## 2. Results and Discussion

### 2.1. Preparation of WCOT Columns

To achieve reproducible results in obtaining robust WCOT columns, the development process is quite laborious. The fused silica capillary should be well treated (physically and chemically) before coating with the stationary phase film. Such processes (pretreatment and silanization) are necessary to ensure separation efficiency and column inertia. Therefore, we need the formation of a homogeneous film and the reduction of secondary adsorption effects of silanol groups, respectively. Separation efficiency and column inertia are concepts related to the film’s formation on the column’s inner surface. That is, the better the column is covered with this liquid film, the less residual silanols will be exposed, and the column inertia will be guaranteed.

**Figure 1:**
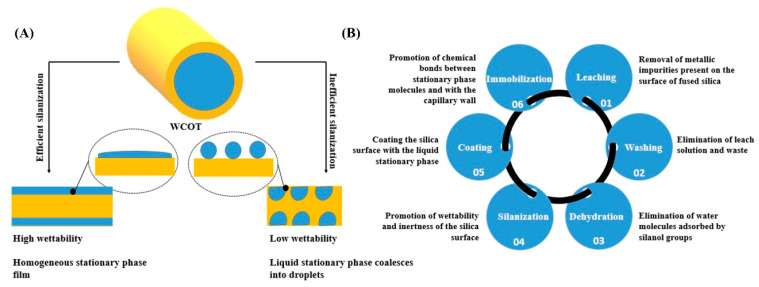
(**A**) The difference in the silica surface’s coating with the liquid stationary phase depends on the wettability profile; (**B**) summary of the steps involved in developing WCOT columns and their respective functions.

To perform a good column coating, the concept of wettability must be considered, which is the ability of a liquid to maintain contact with a solid surface [17]. A homogeneous film is formed on the wall if the surface has high wettability. However, if the surface has low wettability, the liquid tends to agglutinate on the tube wall, and the separation efficiency and inertia will be compromised, generating chromatographic peaks with greater width at the base and with a tail (Figure 1A). One way to promote the silica surface’s wettability is through its treatment with a silanizing agent that contains functional groups similar to those of the liquid stationary phase. In this work, all the columns were silanized with the same agent HMDS (hexamethyldisilazane), which is one of the agents that guarantees more inertia for the columns [18].

Figure 1B presents a systematization of all the steps in preparing WCOT columns and their functions. Briefly, the pretreatment steps remove any traces of metals and/or dirt that may be present in the fused silica capillary. Next, as discussed, the silanization will promote the capillary wall’s wettability to receive the stationary phase film. Furthermore, finally, the formed film will be immobilized on the column wall using a radical primer. This immobilization involves chemical bonds between the stationary phase film and the tube wall and chemical bonds between film molecules.

### 2.2. Screening of Stationary Phases

This work chose five liquid stationary phases to prepare WCOT columns. The choice of phases was guided by the McReynolds constants commonly applied in gas chromatography. Such constants give an idea of the polarity of the stationary phase [19,20]. Table 1 presents the respective McReynolds constants for each chosen phase.

**Figure 2:**
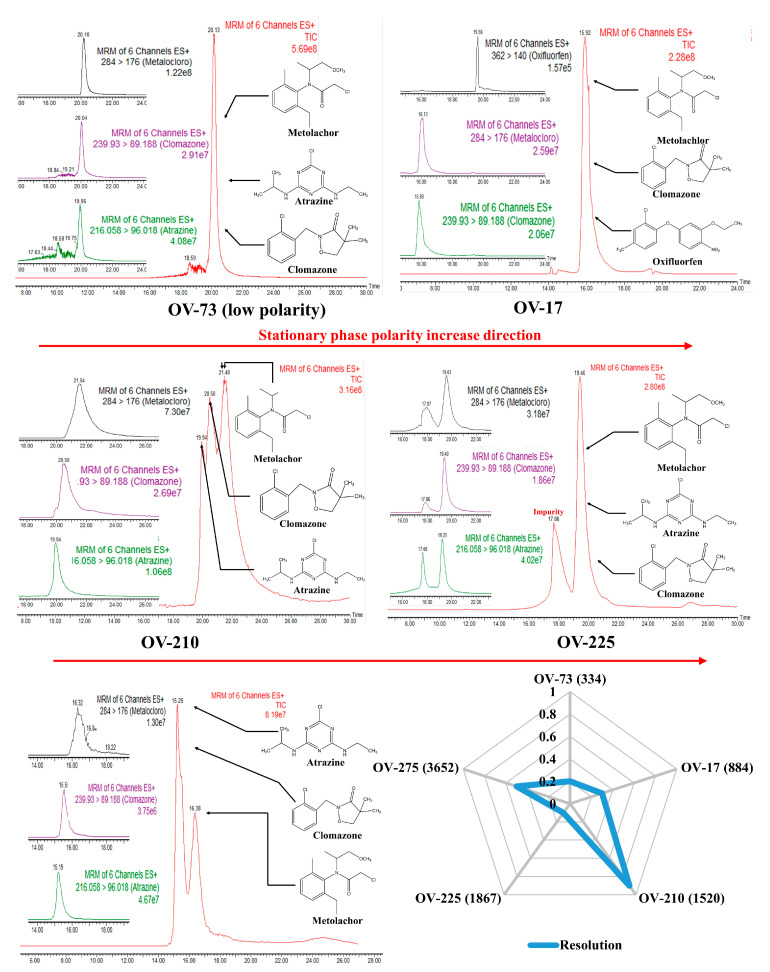
Radar charts and chromatograms obtained for WCOT columns coated with OV-73, OV-17, OV-210, OV-225, and OV-275 stationary phases.

Figure 2 presents a radar plot constructed with chromatographic resolution (Rs) data between clomazone and metolachlor analytes. Furthermore, the total ion chromatograms (TIC) and the respective MRM transitions for each analyte (atrazine, clomazone, and metolachlor) obtained for each stationary phase tested in the work were presented. Finally, selecting the best stationary phase for developing WCOT-LC columns was conducted, considering the Rs and the evaluation of the chromatograms.

The OV-73 column (5% phenyl 95% dimethyl polysiloxane) constitutes a stationary phase of low polarity with a low selectivity due to the presence of mostly methyl groups. This phase is widely used in gas chromatography, but it did not present enough separation for the analytes tested to justify proceeding with OV-73 in liquid chromatography. Similarly, the OV-17 column (50% phenyl, 50% dimethyl polysiloxane), of medium polarity, also did not show chromatographic separation, with the analytes coeluting in one large band. Atrazine did not result in an excellent chromatographic probe for evaluating this column. Therefore, it was changed to oxyfluorfen. Although we could identify each analyte separately from their MRM transitions by mass spectrometry, the same would not happen if another type of detector were used, such as UV-Vis or fluorescence, once their retention times were too close.

**Figure 3:**
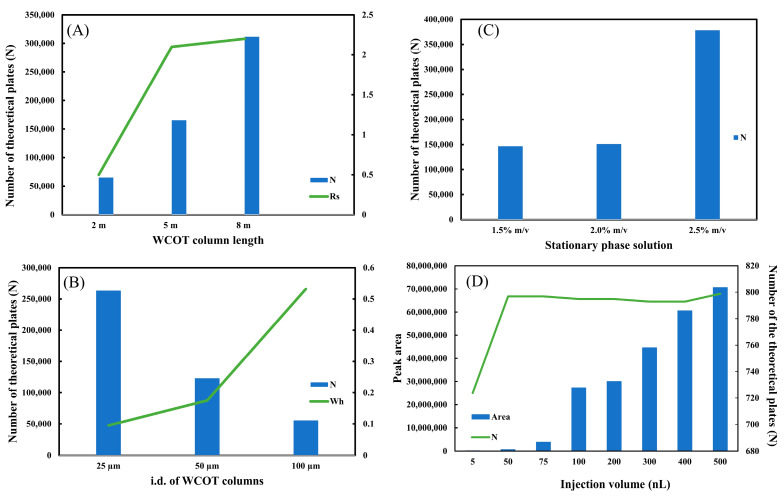
Optimization of WCOT columns. (**A**) Graph of length; (**B**) graph of stationary phase solution; (**C**) graph of inner diameter; (**D**) graph of injection volume. All graphics were constructed with clomazone standard data (500 μg L^−1^).

The OV-210 column (50% trifluoro propyl 50% dimethyl polysiloxane) of medium polarity was the one that presented the best results, among the evaluated columns, to separate the analytical probes selected. Therefore, this phase was selected to proceed with our work on the WCOT-LC columns preparation. As we can see in Figure 3, the selected analytes are separated into three peaks. Despite not being a separation with individual peaks, as would be desired, this was the best column among the evaluated phases. Furthermore, although the MRM transitions have relatively broad peaks, we should consider that this is an experimental column, and it still needed to go through the evaluation stage of the parameters affecting its performance.

The OV-225 column (25% phenyl, 25% cyano, 50% dimethyl polysiloxane) of medium polarity also did not present a chromatographic separation, in addition to observing some “unknown peaks” in the baseline of the chromatogram, which can even be confused with an analyte, but probably corresponds to some impurity in the stationary phase. Precisely for this reason, we disregarded the OV-225 column.

Finally, the higher polarity OV-275 column (100% cyanopropylsiloxane) was tested. The stationary phases with cyano content can operate in reversed-phase and normal-phase chromatography, but the best results are obtained in the last-mentioned way. In Figure 3, we have the OV-275 column being applied in reverse phase chromatography, demonstrating a partial separation since there was a coelution of atrazine with clomazone and separation only of metolachlor. Unfortunately, normal phase chromatography is not a favorable mode for electrospray mass spectrometry since, in this mode, non-polar mobile phases—usually hexane—are commonly used, which are not ionizable. Consequently, there is instability of the MS signal, resulting in low-reproducibility chromatograms. Some attempts were made in our laboratory in this direction, but no successful results were obtained. Therefore, the OV-275 column was also not further considered.

In general, Figure 2 demonstrates that increasing the polarity of the stationary phase increases its selectivity and the separation capacity. Additionally, in reversed-phase liquid chromatography, it is not recommended to use columns of low polarity such as OV-73, as they have much lower selectivity, provoking coelution of the analytes. However, in our experimental conditions it is not appropriate to use high polarity columns such as OV-275, as they are more suitable for normal phase chromatography. Therefore, the best stationary phase composition for WCOT-LC among the investigated in this work is medium polarity phases (OV-17, OV-210, and OV-225), with the best result obtained for OV-210, which was chosen to continue the work. 

### 2.3. Evaluating Parameters That Affect WCOT Columns

The WCOT columns, now with the OV-210 stationary phase, were evaluated to investigate the influence of some parameters on the efficiency of the column. The first variable was the column length. Three lengths (2 m, 5 m, and 8 m) were investigated. In gas chromatography, where WCOT columns are most used, columns of 30 m in length are found, but this length is not yet very suitable in liquid chromatography. Considering that the molecular diffusion of analytes in a liquid medium is slower than in a gaseous medium, too-long columns would result in very long analysis times [21]. Ishii published some work employing octadecylsilane (ODS) coated WCOT columns to separate polycyclic aromatic hydrocarbons. These columns had a length of around 20.8 m [5] and 22.0 m [10]. They required about 90 and 120 min to complete the chromatographic analysis, even using a higher mobile phase flow rate than the one reported in this work.

Figure 3A shows the plot of N and Rs (between clomazone and metolachlor) as a function of length. It is observed that the increase in length leads to an increase in column efficiency, expressed in N. Furthermore, there is a significant gain in separation resolution starting from L = 2 m to L = 5 m. Comparing the Rs for the 5 m and 8 m columns, it is concluded that the separation capacity is practically the same. Therefore, we chose L = 5 m as the most suitable length for WCOT columns. Although L = 8 m has an excellent efficiency (N), its separation capacity (Rs) is similar to L = 5 m. In addition, the greater the column length, the greater the difficulty in experimentally preparing WCOT columns, ensuring homogeneous stationary phase films throughout the column, and increasing the chances of clogging in the column itself or analytical instrumentation. The chromatographic separation data can be consulted in Figure S1, and the numerical Rs and N data in Tables S2 and S3, respectively, in the Supplementary Materials.

The second parameter evaluated was the internal diameter. Similar to the length, three levels of i.d. (25 μm, 50 μm, and 100 μm) were also tested. Figure 3B shows the graph of N and Wh as a function of internal diameter. As predicted by the miniaturization theory in liquid chromatography [1], the smaller the internal diameter, the greater the column efficiency (N). Therefore, the graph demonstrates a fall of N from i.d. 25 μm to i.d. 100 μm. Another interesting piece of data is the width at half height of the peak (Wh). As the diameter increases, the Wh increases significantly, demonstrating an enlargement of the chromatographic band. This band expansion can be consulted in Figure S2. Therefore, the best internal diameter investigated in this work is 25 μm, as we guarantee maximum efficiency (N) with the lowest band widening (Wh).

Then, the stationary phase solution used to coat the WCOT column was evaluated. Three conditions were tested: 1.5%, 2.0%, and 2.5% *m*/*v* of stationary phase solution. The mass amount of the liquid phase employed will directly impact the thickness of the film generated within the column. The thinner the film generated, the more stable it is, but the lower the load capacity of the column, causing possible overloading problems. On the other hand, the thicker the film generated, the less stable it is, but the greater is its loading capacity. Figure 3C plots N versus stationary phase solution. It is observed that the efficiency of a 1.5% and 2.0% *m*/*v* column is very similar. However, when it increases to 2.5% *m*/*v* (=50 mg of stationary phase), there is a significant increase in N. For WCOT columns, the 2.5% *m*/*v* solution was chosen. Unfortunately, we still cannot estimate the thickness of the film generated in the column under the experimental conditions employed. Usually, PLOT columns that have a porous film on their inner wall can be analyzed by scanning electron microscopy (SEM), being able to estimate the generated film. However, WCOT columns have a liquid film on their wall, which is not visible by SEM, making it more difficult to determine the film thickness. Therefore, for future research within the WCOT-LC theme, it would be interesting to seek a characterization technique for these films.

The injection volume was also studied at this stage. As previously described, columns with thin films have a lower load capacity and a greater possibility of overloading. Therefore, a potential source of column loading is the sample injection volume. Figure 3D shows the plot of N and peak area as a function of injection volume.

The data suggest that injection between 100–500 nL provides a similar efficiency (N), with the peak area value proportional to the injection volume. However, the efficiency is lower under low injection volumes (5 to 75 nL). Furthermore, very low volumes may be outside the commercially available chromatograph’s accuracy limit, compromising data reproducibility. Therefore, the optimal working range for the WCOT-LC columns produced is 100–500 nL. Nevertheless, to preserve the column and avoid clogging due to the low load capacity of these columns, the most ideal would be to work with 100 nL.

### 2.4. Performance Evaluation: Knox and Poppe Graphics

**Figure 4:**
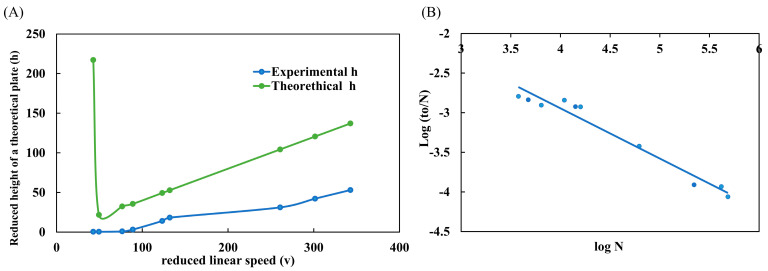
Performance evaluation of WCOT columns. (**A**) Knox plot (h/v) comparing experimental and theoretical curves; (**B**) Poppe plot (log to/N × log N) demonstrating the speed-efficiency relationship. Both graphs were constructed with the standard clomazone data (500 μg L^−1^).

**Figure 5:**
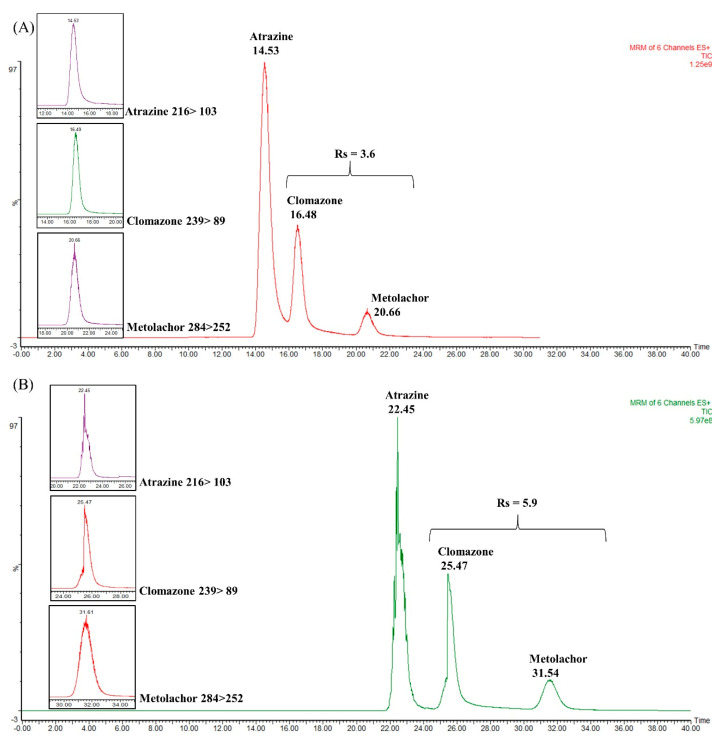
Total ion chromatograms (TIC) obtained in the coupling between WCOT column and packed trapping column to separate the atrazine, clomazone, and metolachlor mixture. (**A**) TIC obtained for commercial trapping column (2 cm × 180 μm i.d. × 5 μm), with Rs = 3.6; (**B**) TIC obtained by coupling the commercial trapping column and a WCOT (5 m × 25 μm i.d. × 2.5% *m*/*v* OV-210) with Rs = 5.9.

One of the main graphs used to evaluate the performance of a column is the H/*u_o_* graph, also known as the Van Deemter curve [22]. This graph allows us to conclude the best linear velocity (*u_o_*) for a given column that provides the maximum efficiency (H). In the graph, this best condition is found on the lowest slope of the curve. However, the Van Deemter curve is commonly employed to analyze LC-packed columns. Therefore, the Knox curve should be used to better analyze WCOT-type capillary columns in micro and nanoLC scales. The graph is very similar to Van Deemter’s, with the difference of applying reduced parameters (h/*v*), which are dimensionless. The form of interpretation is also the same. In Figure 4A, the experimental data of the reduced height of a theoretical plate (h) was plotted as a function of the reduced linear velocity (*v*). Additionally, in Figure 5A, we can compare the experimental and theoretical curves. The experimental data follow the same trend as the theoretical curve. However, the experimental data do not have a maximum h (as observed in the theoretical curve). This occurs because this region of the graph represents the A term, which characterizes the diffusion of analytes due to multiple paths in a packed column. As open tubular columns have a “hollow interior”, they do not suffer this effect (A term = 0), and therefore, there is no such region in the experimental data. Therefore, from the Knox plot, we can conclude that the best *v* range is between 50–100, which experimentally corresponds to a mobile phase flow rate between 100–175 nL min^−1^.

Poppe’s graph was also plotted with the same data as Knox’s graph. Poppe brings a good idea about the speed-efficiency relationship [23,24]. In Figure 4B, we plot log to/N as a function of log N. From the graph, we understand that we need slower analyses (greater log N) to obtain maximum efficiency (greater log to/N). On the other hand, to obtain faster analyses (lower log to/N), the column efficiency is sacrificed (lower log N). This conclusion was visualized throughout all work with WCOT columns. If we used fast gradients, the chromatographic peaks were narrower (high efficiency) but with low separation (practically coeluted). Moreover, if we used slower gradients, there would be an enlargement of the chromatographic peaks (loss of efficiency) but with separation.

Poppe’s graph satisfactorily demonstrates the speed-efficiency relationship of columns and corroborates the experimental observations obtained in the research.

### 2.5. Coupling WCOT Column with a Trapping Column

The last step of this work was the coupling of the produced WCOT-LC column (5 m × 25 μm i.d. × coating with 2.5% *m*/*v* OV-210) with a packed trapping column (C18 Symmetry 2 cm × 180 μm i.d. × 5 μm). The purpose was to observe how this configuration could impact the WCOT separation capacity. Initially, the trapping column was connected to an empty fused silica tube (1 m × 25 μm i.d.) to verify its ability to separate the target analytes (atrazine, clomazone, and metolachlor). Next, the materials and methods item described the loading conditions and chromatographic separation employed.

The optimization of the sample loading step in the trapping column was performed manually, observing the chromatographic results. Therefore, the first parameter evaluated was the loading time. Initially, 1 min of loading was tested, and the results proved that this was an insufficient time for loading. Thus, the time was increased to 3 min, which proved adequate. The second parameter evaluated was the flow rate of the mobile phase during the elution step (valve in position 2). In this position, the mobile phase passes through the trapping column, eluting the analytes to the WCOT column, where chromatographic separation will occur. As judged by the Knox curve, the optimal flow rate for the WCOT column is between 100–175 nL min^−1^. Therefore, we tested a flow rate of 100 nL min^−1^ in the elution step. The results demonstrate that this flow was too low to remove analytes from the 5 μm particle-packed trapping column rapidly. According to Knox’s plots, for injections into the WCOT column without a trap column, it might be the optimal flow rate. However, higher flows capable of eluting the analytes from a packed bed are required for this application. Therefore, 300 nL min^−1^ was tested. This flow rate was sufficient to elute the analytes.

**Figure 6:**
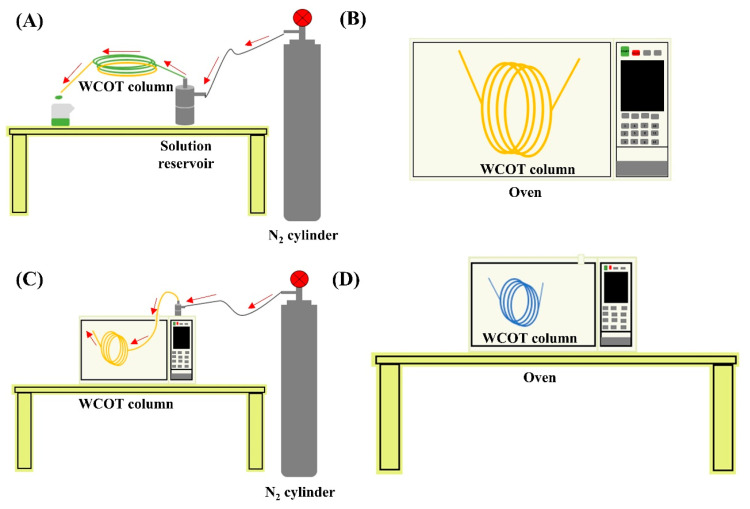
WCOT columns preparation system; (**A**) instrumentation for leaching, solvent washing, and column coating; (**B**) drying step under constant temperature; (**C**) instrumentation for column dehydration process under constant N_2_ flow; (**D**) stationary phase film formation step after column coating. The red arrows indicate the path taken by the flow of N_2_.

Figure 5A shows the chromatogram obtained with the single commercial trapping column. As can be seen, the analytes are separated, especially the metolachlor that has a higher retention time. Atrazine and clomazone are still not very well separated at their baseline. Figure 6B shows the chromatogram corresponding to the coupling between the C18 symmetry column and the WCOT. The result demonstrates a better separation between atrazine and clomazone. In addition, the retention times were extended, as the WCOT column has 5 m.

Regarding chromatographic resolution (between clomazone and metolachlor), we started from Rs = 3.6 in the trapping column to Rs = 5.9 in the WCOT column. Therefore, the use of trapping columns together with WCOT columns can be an approach to be explored in future works since, under this configuration, there was an improvement in the separation (visualized between atrazine and clomazone). However, more efforts are required to optimize WCOT columns (preparation and chromatographic conditions) to become competitive columns similar to LC-packed columns in the future.

## 3. Review of Similar Works

Table 2 summarizes some works reported in the literature on developing WCOT columns applied to LC. As we can see, most of the work was carried out during the 1980s and 1990s, reawakening the scientific community’s interest only in 2019, with the development of columns with 2 μm i.d. In Table 3, we see a consensus among works in employing stationary phases of ODS (octadecylsilane) that do not have much selectivity, and the analytical standards are, in their majority, based on PAHs or anthracene derivatives (also a PAH). Furthermore, all analyses were performed on conventional LC-UV-Vis. However, these works (i) do not explain how flows in the μL min^−1^ range are achieved in conventional LC systems; (ii) do not detail the column preparation system used to produce columns with i.d. < 10 μm; and do not present a systematic study to evaluate relevant parameters that may affect column efficiency. Therefore, here lies the difference in our work. In the present work, a step-by-step optimized methodology is used for preparing LC-WCOT columns with stationary phases based on various dimethyl polysiloxane, commonly applied only in gas chromatography. Moreover, the proposed work is supported by a systematic study of the parameters that affect column efficiency. Another differential of our work is the application of WCOT columns prepared with a liquid stationary phase based on dimethyl polysiloxane in nanoLC-ESI-MS/MS systems, with more polar analytes, which deviate from the commonly-used PAHs analysis.

## 4. Materials and Methods

### 4.1. Analytical Standards and Reagents

High purity (>99%) analytical standards of clomazone, atrazine, metolachlor, and oxyfluorfen were obtained from Fluka Analytical (St. Louis, MO, USA). Stock solutions were prepared by diluting the standard in methanol to a concentration of 1000 mg L^−1^ and stored in amber bottles at −7 °C. Working solutions were prepared by diluting stock solutions to the concentrations of interest. High purity (>99%) solvents used as the mobile phase were acetonitrile, acquired from Tedia (Fairfield, CT, USA), and water from Merck Millipore Ultrapure Water System (Burlington, VT, USA). Formic acid used as an additive to the mobile phase was purchased from Sigma-Aldrich (St. Louis, MO, USA).

### 4.2. Chromatographic Apparatus and Analytical Conditions

The WCOT columns were evaluated in a UPLC system consisting of an ACQUITY UPLC liquid chromatograph from Waters (Milford, CT, USA), equipped with an ACQUITY UPLC sample manager and binary solvent pumps, coupled to the XEVO TQ MS tandem mass spectrometer with nano electrospray ionization. Chromatographic analyses were performed in a partial loop (1 μL), injecting a volume of 500 nL for a concentration of 500 μg L^−1^, with temperature and flow rate of 21 °C and 300 nL min^−1^, respectively.

The mass spectrometry detection method was obtained by IntelliStart software (4.1) (XEVO TQ MS optimization tool) in positive ionization mode (ESI+) and multiple reaction monitoring (MRM). For this purpose, each analyte was introduced into the MS/MS by direct infusion at a concentration of 0.5 μg L^−1^. Furthermore, two transitions were chosen to identify these compounds (Table 3). The calibration conditions (“tunning”) for the nano-ESI used in the chromatographic analyzes consisted of capillary and cone voltages of 3.5 kV and 30 V, respectively, and the source temperature was 100 °C.

### 4.3. Preparation of WCOT Columns

WCOT columns were prepared with fused silica tubes of 50 μm internal diameter and 375 μm outside diameter, purchased from Polymicro Technologies (Phoenix, AZ, USA). Peek sleeves, washers, and ferrules necessary for connecting the column to the UPLC-MS/MS system were purchased from Acore (Sao Paulo, Brazil). The development of WCOT columns can be subdivided into the following stages: tube pretreatment, silanization, stationary phase coating, and film immobilization (cross-linking).

#### 4.3.1. Tube Pretreatment

The fused silica tubing was leached with 2% *v*/*v* HCl solution from Tedia (Fairfield, CT, USA) under nitrogen flow at 80 bar for 5 min. Then, it was heated in an oven at 220 °C for 3 h. Subsequently, the capillary was washed with 1.5 mL of the same hydrochloric acid solution and 1.5 mL of dichloromethane. Afterward, it was dried with nitrogen gas for 30 min. To complete the pretreatment, the column was subjected to a dehydration step in which it was heated at 200 °C for 2 h under a continuous flow of nitrogen gas at 40 bar.

#### 4.3.2. Silanization

After completion of column pretreatment, the capillary was directed to the silanization process with hexamethyldisilazane (HMDS) from Sigma Aldrich (St. Louis, MO, USA). This process consists of the percolation of the silanizing agent for 5 min through the capillary, under nitrogen flow at 80 bar, followed by heating in an oven at 290 °C for 6 h.

#### 4.3.3. Stationary Phase Coating

The column coating was carried out by percolating the stationary phase solution for 30 min under nitrogen flow at 80 bar. Subsequently, the column was heated in an oven at 40 °C for 15 min. The stationary phase solution (2% *m*/*v* solution) was previously prepared by dissolving 40 mg of the liquid phase in 2 mL of a 1:1 dichloromethane/pentane solution.

#### 4.3.4. Film Immobilization

The stationary phase film formed inside the column was immobilized with the azo-tert-butane (ATB) radical initiator obtained from Sigma Aldrich (St. Louis, MO, USA). For this purpose, the ATB was percolated through the column for 5 min and then heated in an oven at 220 °C for 1 h. This process was repeated three times. Figure 6 presents the instrumental part of the development of the columns.

### 4.4. Screening of Stationary Phases

Five liquid stationary phases were tested to separate the analytical standards mentioned in Section 2.1. OV-73 (5% phenyl 95% DMPS); OV-17 (50% phenyl 50% DMPS); OV-210 (50% trifluoro propyl 50% DMPS—dimethylpolysiloxane); OV-225 (25% cyano 25% phenyl 50% DMPS); and OV-275 (100% cyanopropyl DMPS) were obtained from the Ohio Valley Specialty Company (Marietta, OH, USA). The choice of phases was based on the McReynolds constant, which gives an idea of the polarity of the stationary phase. The screening was performed by injecting a mixture of atrazine, clomazone, and metolachlor (500 μg L^−1^) under gradient conditions. Separation resolution data between clomazone and metolachlor analytes (which are the most retained) were computed and plotted on a radar chart.

### 4.5. Evaluating Parameters That Affect WCOT Columns

After choosing the best stationary phase within the experimental conditions, some parameters that directly affect the efficiency of a column were evaluated. Thus, column length, internal diameter, and film thickness were studied at three different levels for each parameter mentioned (low, medium, and high level). Other parameters, such as mobile phase flow rate, injection volume, and coupling with the trapping column, were evaluated to provide the best operating conditions for the WCOT.

#### 4.5.1. WCOT Column Length

Lengths of 2 m, 5 m, and 8 m were evaluated to determine the most appropriate for our WCOT columns. The other parameters referring to the column remained constant, 50 μm i.d. and stationary phase (2% *m*/*v* solution). The number of plates (N) and separation resolution (Rs) data were computed in this experiment, according to Table S1.

#### 4.5.2. WCOT Column Inner Diameter

Three internal diameters were evaluated: 25 μm, 50 μm, and 100 μm, keeping the other column parameters constant. The other parameters referring to the column remained the same (5 m of length and stationary phase coating with 2% *m*/*v* solution). The number of plates (N) and width at half height (Wh) data of the chromatographic peak were obtained for this experiment.

#### 4.5.3. Stationary Phase Mass in the Coating

The mass amount of liquid stationary phase used to coat the column directly affects the thickness of the film generated in the column and, consequently, its separation efficiency. Thus, 1.5%, 2%, and 2.5% *m*/*v* of the stationary phase (=30 mg, 40 mg, and 50 mg of the liquid phase in 2 mL of solvent) were tested to determine the most efficient. The other parameters referring to the column remained constant; 25 μm i.d. and 5 m long. N data were computed as well.

#### 4.5.4. Optimum Flow Rate of the Mobile Phase

The optimal flow for the WCOT column was evaluated by injecting the analytical standard of clomazone (500 μg L^−1^ and 500 nL of injection volume) in a mobile phase flow range of 100–800 nL min^−1^. Then, data processing involved plotting the reduced height of the theoretical plate (h) as a function of the reduced linear velocity (*v*). The equations used for the calculations are in Table S1 in the Supplementary Materials. The molecular diffusion of the analytes in the reversed-phase (Dm = 1.10^−5^ cm^2^ s^−1^) was used in the calculations.

#### 4.5.5. Optimum Injection Volume

Injection volume assessment for WCOT columns was assessed by injecting the clomazone (500 μg L^−1^) over a 5–500 nL range in partial loop mode (1 μL capacity). The peak area and the number of plates were computed.

#### 4.5.6. Coupling WCOT Column with a Trapping Column

We also evaluate the coupling of a WCOT column to a “trapping” C18 Symmetry column (2 m × 180 μm i.d. × 5 μm, Waters—Milford, CT, USA) through switching valves. Two valves were used for this purpose. In the first moment, which we call position 1, the loop is filled with the sample (in valve 1) and then loaded into the packed trapping column (in valve 2), with the loading mobile phase, which also passes through the WCOT analytical column. In a second moment, which we call position 2, the analytes elute from the trapping column to the WCOT column, with the elution mobile phase, under gradient conditions.

The loading step (when analytes are eluted to the trapping column) was evaluated for 1–3 min, with a mobile phase flow rate of 8 μL min^−1^ and conditions of 99% H_2_O (A) and 1% ACN (B) plus 0.1% formic acid. The elution step (when analytes are eluted from the trapping column to the WCOT) was evaluated for a flow between 100–300 nL min^−1^, under gradient conditions: 0–0.1 min with 1% B, 0.1–0.5 min increases to 60% B and holds up to 21 min, 21–51 min return to 1% B to recondition the WCOT column.

## 5. Conclusions

The work reported here presented the development and evaluation of selected parameters that affect WCOT-type open tubular columns in nano-LC-ESI-MS/MS systems. The results after all evaluations of some parameters that affect WCOT open tubular columns of stationary phase, length, internal diameter, and optimal flow rate, among others, and the application of the column in line with a trapping column, proved to be satisfactory, considering the limitations—particularly in the instrumentation side—and difficulties of the work. The WCOT column alone presents separation capability, as seen in Figure S1, and under coupling conditions with a trapping column (Figure 6B), it presents the potential to be a new column possibility for LC systems. However, there is still much to study about WCOT columns in LC, as we still face limitations such as liquid film detachment, which leads to column and chromatographic system clogging. In short, this work aimed to show the potential of WCOT columns as a future trend in LC, which still requires greater attention from the scientific community to overcome its limitations. We hope that it will stimulate other research groups to be involved with the theme, thus bringing the WCOT to its deserved position in the top LC columns, as anticipated long ago by the chromatography theory.

## Figures and Tables

**Table 1:** Chemical composition and McReynolds constants of the stationary phases tested in the coating of WCOT columns.

n°	Composition	Stationary Phase	McReynolds Constants
1	5% phenyl 95% DMPS	OV-73	334
2	50% phenyl 50% DMPS	OV-17	884
3	50% trifluoropropyl 50% DMPS	OV-210	1520
4	25% cyano 25% phenyl 50% DMPS	OV-225	1867
5	100% cyanopropyl DMPS	OV-275	3652
	DMPS: dimethylpolysiloxane		

**Table 2:** WCOT application reports found in the literature.

n°	Stationary Phase	Column Dimension	Analytes	Instrument	Flow Rate	Time	Elution	Year
1	ODS *	3.84 m × 60 μm	Benzene, Naphthalene, Biphenyl	LC-UV	2.2 μL/min	20 min	25:75 ACN/H_2_O	1978
2	ODS	20.80 m × 56 μm	Benzene, Biphenyl, and HPAs	LC-UV	1.0 μL/min	90 min	40:60 ACN/H_2_O	1978
3	ODS	6.00 m × 100 μm	Resorcinol, Naphthalene, Anthracene	LC-UV	1.1 μL/min	90 min	40:60 ACN/H_2_O	1980
4	ODS	5.30 m × 38 μm	Indole-3-acetic acid	LC-UV	1.1 μL/min	12 min	10:90 ACN/acetic acid aqueous solution	1983
5	ODS	22.00 m × 31 μm	HPAs	LC-UV	0.52 μL/min	120 min	ramp from 40 to 70% ACN	1983
6	PS-255 *	2.08 m × 11.6 μm	Anthracene derivatives	LC-UV	0.022 μL/min	40 min	40:60 ACN/H_2_O	1987
7	PS-255	1.93 m × 11.3 μm	Anthracene derivatives	LC-UV	not reported	2 min	70: 30 ACN/H_2_O	1987
8	PMSC_18_ *	1.44 m × 5.8 μm	Anthracene derivatives	LC-UV	0.013 μL/min	4 min	50:50 ACN/phosphate aqueous solution	1991
9	PMSC_18_	5.80 m × 6.3 μm	Anthracene derivatives	LC-UV	0.0046 μL/min	130 min	50:50 ACN/H_2_O	1993
10	ODS	0.80 m × 2.0 μm	Glycine, Isoleucine, Leucine	LC-UV	18 μL/min	7 min	20:80 ACN/ammonium aqueous solution	2019
11	OV-210	5.00 m × 25 μm	Atrazine, Clomazone, Metolachlor	nanoLC-ESI-MS/MS	0.30 μL/min	60 min	Gradient ACN/H_2_O	This work

ODS * = octadecylsiloxane/PS-255 * = 1% 1% vinyl/99% DMPS/PMSC_18_ * = polymethyloctadecylsiloxane/OV-210 * = 50% trifluoropropyl/50% DMPS.

**Table 3:** Optimization of the transitions obtained with the help of the IntelliStart Software (Waters) for the target analytes and their respective cone voltages and collision energy.

Analyte	MM	log P	Precursor Ion (*m*/*z*)	Product Ion (*m*/*z*)	Dwell (s)	Cone (V)	Collision Energy (V)
Atrazine	215.68	2.61	216.06	96.018	0.512	22	20
				103.95	0.512	22	26
Clomazone	239.70	2.50	239.93	89.19	0.152	50	46
				124.96	0.152	50	16
Metolachor	283.79	3.13	284.00	176.00	0.152	20	30
				252.00	0.152	20	20
Oxyfluorfen	361.70	4.73	362.00	140.00	0.152	30	50
				2370.00	0.152	20	20

## Data Availability

The corresponding author’s data supporting this study’s findings are available upon reasonable request.

## Supplementary Materials

The following supporting information can be downloaded at: https://www.mdpi.com/article/10.3390/molecules28135103/s1, Figure S1: Total ion chromatogram obtained from the separation of atrazine, clomazone and metolachor in different WCOT column lengths. (A) L = 2 m. (B) L = 5 m. (C) L = 8 m, Figure S2: Peak broadening for each WCOT column internal diameter, obtained in isocratic mode (80:20 ACN/H2O with 0.1% formic acid) for clomazone (500 μg L^−1^), Table S1: Equations used for analysis of WCOT columns, and parameter nomenclature, Table S2: Chromatographic resolution data according to WCOT column length, Table S3: Data on the number of theoretical plates obtained for the WCOT column according to length.

## Sample Availability

Samples of the compounds are not available from the authors.
