# Supplementary material for: Development of Wall-Coated Open Tubular Columns and Their Application to Nano Liquid Chromatography Coupled to Tandem Mass Spectrometry
Source: Molecules. 2023 Jun 29;28(13):5103. doi: 10.3390/molecules28135103 (PMC10343200; doi:10.3390/molecules28135103)

Supplementary information

Development of wall-coated open tubular columns and their application to nano liquid chromatography coupled to tandem mass spectrometry

Natalia G. P. Santos, Deyber A. V. Medina and Fernando M. Lanças *

São Carlos Institute of Chemistry, University of São Paulo, São Carlos 13566-590, Brazil

* Correspondence: flancas@iqsc.usp.br

Table S1. Equations used for analysis of WCOT columns, and parameter nomenclature.

**Equation**	**Parameter nomenclature**
Separation resolution<br>$$R_s = \frac{tR_2 - tR_1}{\frac{(wb_1 + wb_2)}{2}}$$<br>Height equivalent of a theoretical plate<br>$$H = \frac{L}{N}$$<br>Reduced height for a theoretical plate<br>$$h = \frac{H}{N.dc}$$<br>Mobile phase linear speed<br>$$u_o = \frac{L}{tM}$$<br>Reduced linear speed<br>$$v = \frac{u_o.dc}{Dm}$$<br>Number of theoretical plates<br>$$N = 5{,}54\left(\frac{tR}{wh}\right)^2$$	t_R = retention time (s)<br>t_M = dead time (s)<br>Wb = chromatographic peak base width (s)<br>wh = width at half height of the chromatographic peak (s)<br>L = column length (cm)<br>N = number of theoretical plates (-)<br>dc = column inner diameter (cm)<br>Dm = molecular diffusion of analytes in FM (cm^2/s)<br>Rs = separation resolution (-)<br>H = height plate (cm)<br>h = reduced height plate (-)<br>u_o = mobile phase linear speed (cm/s)<br>v = reduced linear speed (-)<br>N = number of theoretical plates (-)

Figure S1. Total ion chromatogram obtained from the separation of atrazine, clomazone and metolachor in different WCOT column lengths. (A) L = 2 m. (B) L = 5 m. (C) L = 8 m.

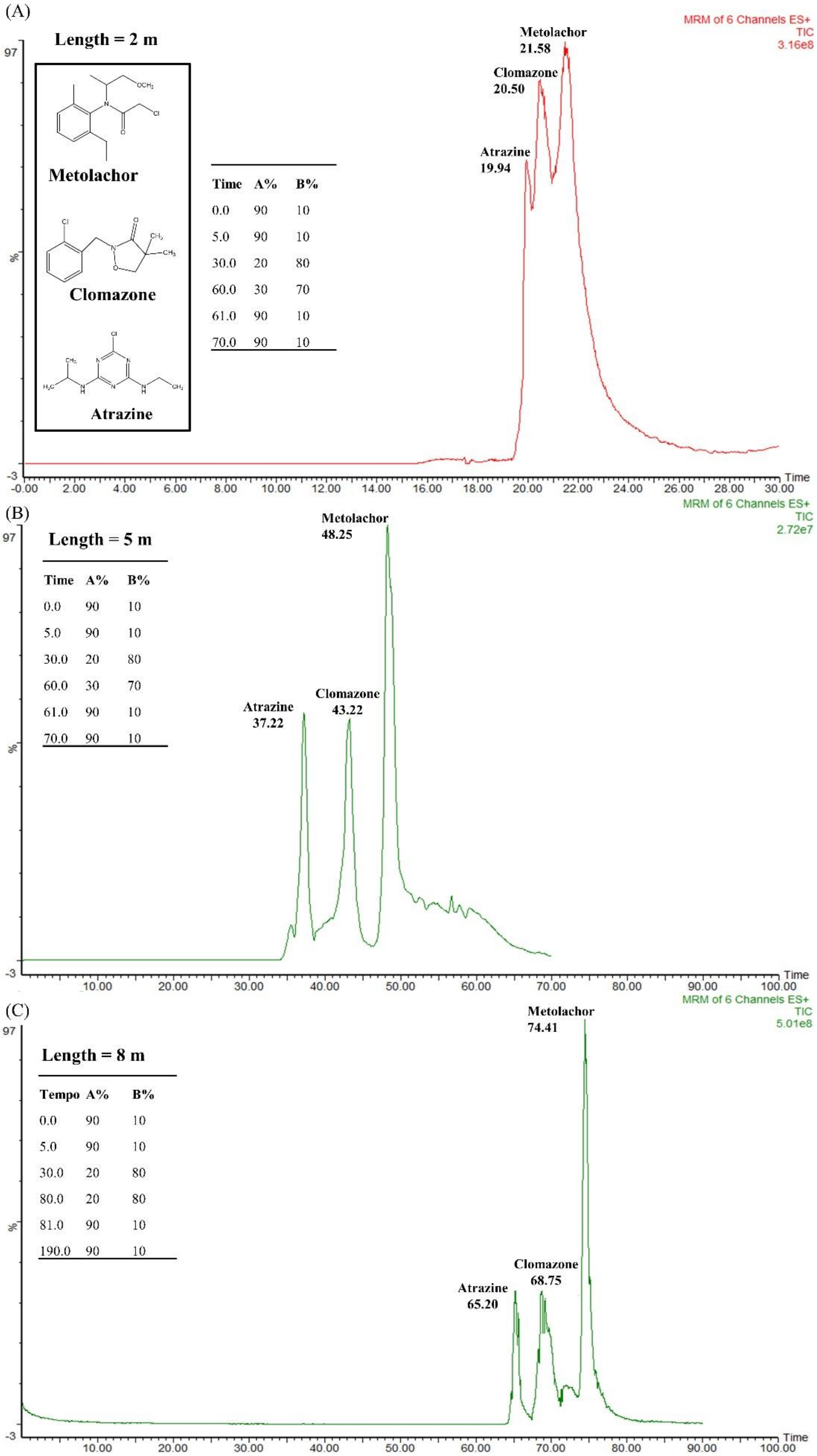

Table S2. Chromatographic resolution data according to WCOT column length.

Length	tR (A)	tR (C)	tR (M)	Wb (A)	Wb (C)	Wb (M)	Rs*
2 m	19.94	20.50	21.48	1.26	1.92	2.16	0.5
5 m	37.62	42.32	48.15	2.07	2.39	2.62	2.1
8 m	65.20	68.74	74.48	1.65	3.04	2.42	2.2

A: atrazine/ C: clomazone/ M: metolachor/ Rs*: between clomazone and metolachor/ t_R (min); Wb (min)

Table S3. Data on the number of theoretical plates obtained for the WCOT column according to length.

Length	tR (C)	Wh (C)	N*	N/m
2 m	20.50	0.189	65.176	32.588
5 m	38.16	0.221	165.173	33.035
8 m	56.91	0.240	311.504	38.938

N*: data calculated for clomazone (500 µg L^{-1}) under isocratic conditions of 80:20 ACN/H_2O plus 0.1% formic acid/ t_R (min); Wh (min)

Figure S2. Peak broadening for each WCOT column internal diameter, obtained in isocratic mode (80:20 ACN/H_2O with 0.1% formic acid) for clomazone (500 µg L^{-1}).

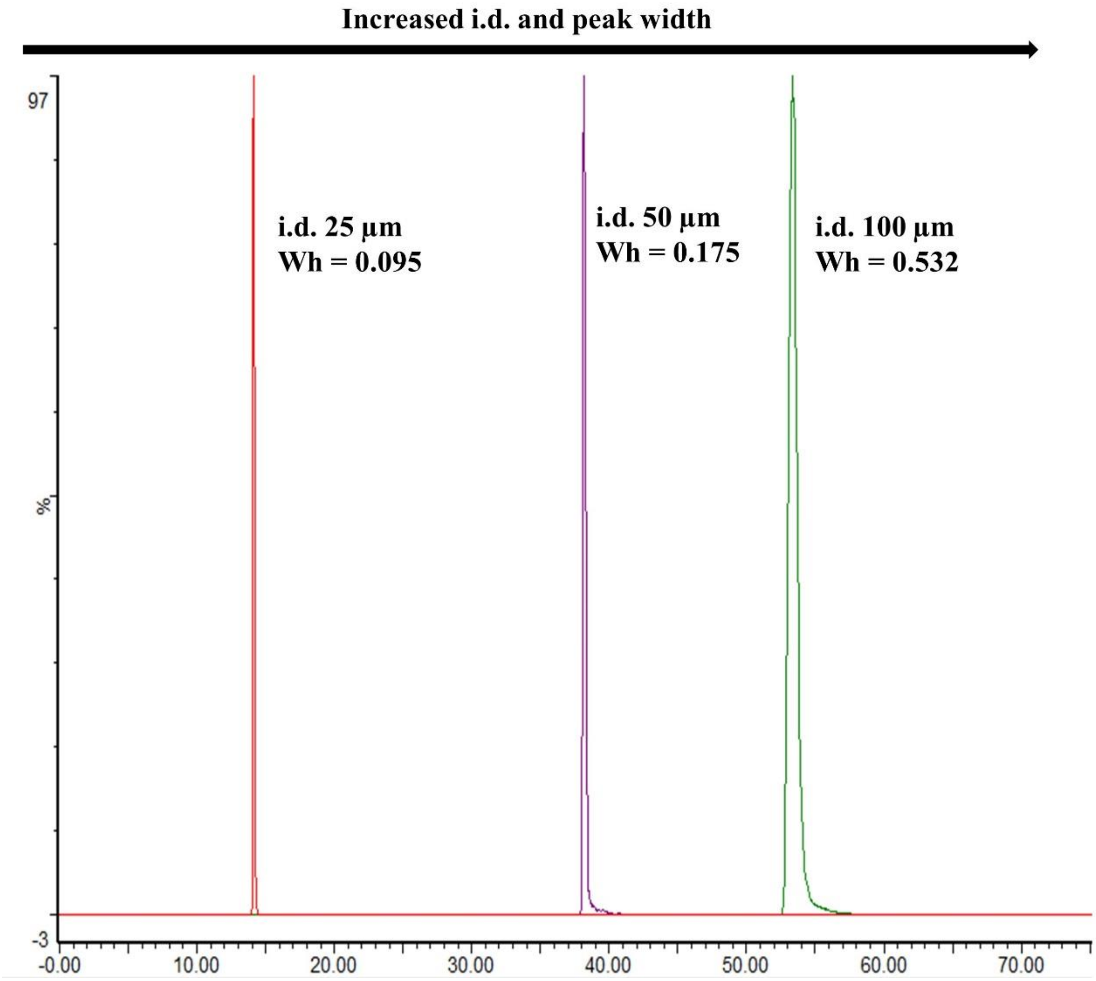

Supplement: Supplementary file 1 [file molecules-28-05103-s001.zip › molecules-2463362-supplementary.pdf]
